# Propolis Integration Methods into Solutions for Highly Loaded Propolis Fibers by Needleless Electrospinning

**DOI:** 10.3390/molecules27072311

**Published:** 2022-04-02

**Authors:** Zane Zelca, Silvija Kukle, Sarmite Janceva, Laimdota Vilcena

**Affiliations:** 1 Institute of Design Technology, Faculty of Materials Science and Applied Chemistry, Riga Technical University, Kipsala Street 6, LV-1084 Riga, Latvia; silvija.kukle@rtu.lv (S.K.); laimdota.vilcena@rtu.lv (L.V.); 2 Latvian State Institute of Wood Chemistry, Dzerbenes Street 27, LV-1006 Riga, Latvia; sarmite.janceva@inbox.lv

**Keywords:** electrospinning, nano and micro fibers, propolis, polyvinyl alcohol, flavonoids

## Abstract

A load-bearing matrix filled with biologically active compounds is an efficient method for transporting them to the target location. Bee-made propolis has long been known as a natural product with antibacterial and antiviral, anti-inflammatory, antifungal properties, and anti-oxidative activity. The aim of the research is to obtain stable propolis/PVA solutions and produce fibers by electrospinning. To increase propolis content in fibers as much as possible, various types of propolis extracts were used. As a result of the research, micro- and nano-fiber webs were obtained, the possible use of which have biomedical and bioprotective applications. All used materials are edible and safe for humans, and fiber webs were prepared without using any toxic agent. This strategy overcomes propolis processing problems due to limitations to its solubility. The integration of different combinations of extracts allows more than 73 wt% of propolis to be incorporated into the fibers. The spinning solution preparation method was adapted to each type of propolis, and by combining the methods, solutions with different propolis extracts were obtained. Firstly, the total content of flavonoids in the propolis extracts was determined for the assessment and prediction of bioactivity. The properties of the extracts relevant for the preparation of electrospinning solutions were also evaluated. Secondly, the most appropriate choice of PVA molecular weight was made in order not to subject the propolis to too high temperatures (to save resources and not reduce the bioactivity of propolis) during the solution preparation process and to obtain fibers with the smallest possible diameter (for larger surface-to-volume ratios of nanofibers and high porosity). Third, electrospinning solutions were evaluated (viscosity, pH, conductivity and density, shelf life) before and after the addition of propolis to predict the maximum propolis content in the fibers and spinning stability. Each solution combination was spun using a cylindrical type electrode (suitable for industrial production) and tested for a stable electrospinning process. Using adapted solution-mixing sequences, all the obtained solutions were spun stably, and homogeneous fibers were obtained without major defects.

## 1. Introduction

Propolis is a natural material produced by honey bees with antibacterial, antiviral, anti-inflammatory, and antifungal properties and antioxidant activity [1,2,3]. Its composition may vary depending on the region of harvest. Aromatic compounds, flavonoids, and phenolic compounds are the main chemical classes present in propolis [4]. Brazil’s green propolis is currently highly valued for its composition (rich in Artepillin C), and brown and red propolis also has great biological potential [1,5]. The solubility and efficacy of propolis against microorganisms depend on the solvent used. Alcohol extracts contain the highest content of flavonoids, but propolis is also soluble in glycerin and polyethylene glycol and partly soluble in water and other substances. Higher levels of flavonoids predict higher efficacy against microorganisms [6], but the overall combination of components is also important. Propolis is commonly used for the treatment of inflammatory dermal diseases and also shows wound-healing activity [7,8]. Therefore, it is very important to find the best way to add it in order to ensure it effective treatment properties.

Electrospun nanofiber webs are extensively studied in this regard; unlike films, they have a high surface area and porosity, which provide a wide range of applications, including in medical treatments. Polyvinyl alcohol (PVA) has an easy process ability and is used as a base material. The quality of electrospun solutions is a key factor in ensuring a stable spinning process and high-quality fibers. To ensure spin ability, it is important to evaluate the viscosity, electrical conductivity, and pH of the solutions. The processability of PVA spinning solution depends firstly on the concentration and secondly on the molecular weight of the polymer. The upper limit of the PVA concentration is highly dependent on the viscosity of the spun solution [9]. Various substances can be incorporated into PVA fibers, and high loading capacity of drugs and other additives can expand their use.

There are currently no publications on pure electrospun propolis nanofibers, but there are several studies [7,10,11,12,13,14] that reflect propolis integration methods in electrospinning solutions incorporated into the fibers or coated on their surface. The following methods have been reported for the integration of propolis in electrospun nanofibers containing PVA:

- Previous solubilization of propolis extract in water/ethanol mixtures [7] showed that propolis does not dissolve and no fibers are formed;
- Preparation of 10–15% PVA solution in mixtures of water/ethanol [7] ensured that propolis dissolves and fibers with various morphology were formed, some of them also with defects;
- Usage of polyethylene glycol (1–2%) as a solvent to solubilize propolis extract, being further added to the PVA solution [7], allowed propolis to dissolve and the allowed the formation of fibers from the solution;
- Preparation of propolis nanoparticles, which were then added to the polymeric solution of PVA [7], led to the successful formation of fibers;
- PVA was used as a supportive material to accumulate propolis in scaffold, and then electrospun Propolis/PVA scaffolds were crosslinked with glutaraldehyde [10];
- Previous ethanol solubilized propolis solutions (3–20%) were added to the PVA (5–24%) solution [11,12,13,14], fibers were formed with various morphologies, most of them without defects.

A number of studies [15,16,17] have shown that propolis ethanol extract containing electrospun PVA fibers have antibacterial activity against *S. aureus* (3 wt% propolis was added directly into PVA/gelatin patch) [15] (40 wt% of propolis added into spinning solution), low activity against *P. aeruginosa* [16], and antiviral activity (fibers containing 5 wt% propolis extract and cross-linked carboxymethyl starch) [17].

In view of relatively low propolis concentrations in the studies examined above, the purpose of this study was to incorporate as much propolis as possible into the fiber composition to achieve material properties that are important for biomedical and bioprotective applications. In order for the resulting solutions to be used in industrial production, it is important to evaluate their shelf life and stability. It is also important to use solutions that are suitable for electrospinning on an industrial scale. Multicomponent electrospinning solutions rapidly lose their homogeneity; therefore, a roller-type needleless electrospinning machine was used in the study to ensure that the solution is stirred even during spinning.

Electrospinning is a simple and effective way to make nanofibers. Several methods for electrospinning nanofibers on a large scale are available. The shortcomings of jet instability and mutual interference are disadvantages of using multi-needle equipment [18]. During the multi-needle electrospinning process, the solution may stratify in syringes if the spinning takes a long time, so a needleless free-surface-spinning machine Nanospider (Elmarco Company, Liberec, Czech Republic) with a rotating cylindrical electrode is preferred, which provides movement of the solution during the spinning and is designed for electrospinning on a large scale. Electrospinning of the free surface is a multivariate process [19] and is influenced not only by the type of electrode and the composition of spinning solutions used but also by the climatic conditions in the room, which affect the evaporation of the solvent and other spinning parameters.

## 2. Results and Discussion

### 2.1. Propolis Characterization

Previous studies have shown that propolis extract in water/ethanol mixtures does not dissolve but could be dissolved in 10–15% PVA water/ethanol solution and nanofibers could be obtained from this solution [7]. Some other investigations show ethanol solubilized propolis in a range of concentrations from 3 to 20% could be mixed with the 10 to 15% PVA water/ethanol solution and obtain fibers from solutions with various morphology [11,12,13,14]. In this study laboratory made propolis water (PW/BRA Lab) and ethyl alcohol propolis extract (PEx/BRA Lab) were prepared (Section 3.3) and used in view of the fact that the largest amount of spinning solution is water (90–94%), so propolis water is one of the most suitable for integration into the solution. Propolis is only partially soluble in water, and there are limited possibilities to obtain propolis water with the highest propolis content. This study analyzed two propolis water types obtained in a laboratory and two industrially produced with different propolis contents to evaluate their suitability for integration in a spinning solution (Table 1). The combination of different propolis waters makes it possible to obtain fibers with customized properties depending on the planned use. 

The pH of propolis water affects the total pH of the solution and may result from the effect of fiber diameters, a slight decrease at a pH of 2 due to the decrease in viscosity in an acidic environment [9]. As can be seen from Table 1, the pH of all four propolis water samples are in range from 3.4 to 4.6.

As part of this study, it has been found that the density of propolis water affects the total viscosity of the solution. The obtained data indicate that the highest density is for propolis water PW/LT (1.01 g/mL), which also contains the highest amount of propolis, and for laboratory-made propolis water obtained from propolis in Brazil and containing Arabic Gum (1.032 g/mL). The increase in density affects not only the total viscosity of the solution but also the mixing time required to dissolve the PVA. Arabic Gum (Gum Acacia) is used as a stabilizer and a thickener in the food and pharmaceutic industries; therefore, an effect on density was expected. Arabic Gum is also useful for PVA film blends tensile properties improvement [20].

The propolis water added to the electrospinning solution effect on electrical conductivity is significant. The conductivity of propolis water samples was in a range from 352 to 1526 µS/cm. A too-high electrical conductivity (up to 1000 µS/cm) can result in an orientation of fibers perpendicular to the support material. As shown in Table 1, PW/LT and PW/BRA Lab samples have high conductivity, but it can be balanced by the addition of low-conductivity propolis extracts in the spinning solution.

High propolis content does not ensure high flavonoid content in propolis water. As shown in Table 1, PW/BRA Lab propolis contains the highest concentration of flavonoids (1.25 mg/g), but its propolis content is only 10%, in comparison with the PW/LT sample, which contains 30.8% propolis but only 0.087 mg/g of flavonoids. This can be affected by both the method of propolis water preparation and the location and time of propolis collection [1]. Comparing the proportions of propolis and flavonoids in aqueous solution and powder, it can be seen that propolis powder (PPa/BRA) has a high solubility in water by boiling, according to the mixing scheme shown in Section 3.3, because propolis powder (~100%) contains 5.43 mg/g of flavonoid, but 10% propolis water (PW/BRA Lab) 1.25 mg/g of flavonoids (Table 1 and Table 2). The sample PW/BRA Lab has the highest concentration of flavonoids in proportion to the propolis content compared to other water samples of propolis–0.0139 mg/g.

Compared with propolis waters, hydroglyceric propolis extract (20–25%) has a higher density (1.220 g/mL) and low electrical conductivity (3 µS/cm), and flavonoid content (1.15 mg/g) is similar to PW/BRA Lab (1.25 mg/g), Table 2. Due to the low electrical conductivity, hydroglyceric propolis extract is suitable for combination with propolis water, which has high electrical conductivity, but care should be taken not to increase the viscosity too much—it is better to use lower PVA concentrations. Glycerol acts as a plasticizer for PVA [21]; it can promote a more even mixing between the water-insoluble part of propolis and affect the mechanical properties of the fiber web.

Due to the very high molecular complex composition of propolis, different extractive processes are needed to remove the inert material and preserve the polyphenolic fraction-flavonoids and phenolic acids. Ethanolic extraction is commonly used for this purpose [6]. The pH of ethyl alcohol propolis extracts is more alkaline than that of propolis water, and no significant effect on fiber diameters is expected due to the excessively acidic environment in electrospinning solutions. Rapid reductions in viscosity can only be observed by adding high levels of ethyl alcohol extracts in low-concentration PVA spinning solutions due to the density rising from 0.885 to 0.905 g/mL (Table 3). Depending on the concentration of PVA, the conductivity of the electrospinning solution may decrease slightly. The main problem with the addition of propolis ethyl alcohol to PVA solutions is the formation of propolis agglomerates due to the reduction of alcohol concentration. It can be seen that the use of a higher alcohol concentration improves the content of flavonoids: 98% ethyl alcohol extract contains 8.87 and 7.53 mg/g, respectively, and 70% extract contains 5.75 mg/g (Table 3). The propolis harvested in Latvia (LV) has a higher flavonoids content in its alcohol extract (PEx/LV Lab) than that harvested in Brazil (BRA) when the alcohol extract (PEx/LV Lab) is obtained, but it is not fully comparable to the PEx/BRA Lab extract due to the small amount of Arabic Gum content in the propolis powder. The wax component of LV propolis has been previously removed. The Arabic Gum swells and dissolves in water, and it has the potential to be used as a wound dressing due to its antibacterial and hemostatic activity [22].

### 2.2. PVA/Propolis Extract Solution Characteristic

The 125 kDa and 130 kDa PVA polymers have short mixing time and dissolution temperature (not exceeding 100 °C). Their viscosity and electrical conductivity are also suitable for all tested concentrations (6, 8, and 10 wt%). After the range of properties of PVA colloidal solutions were determined, possible strategies for the integration of propolis were developed and nanowebs for morphology analysis were made.

**Figure 1:**
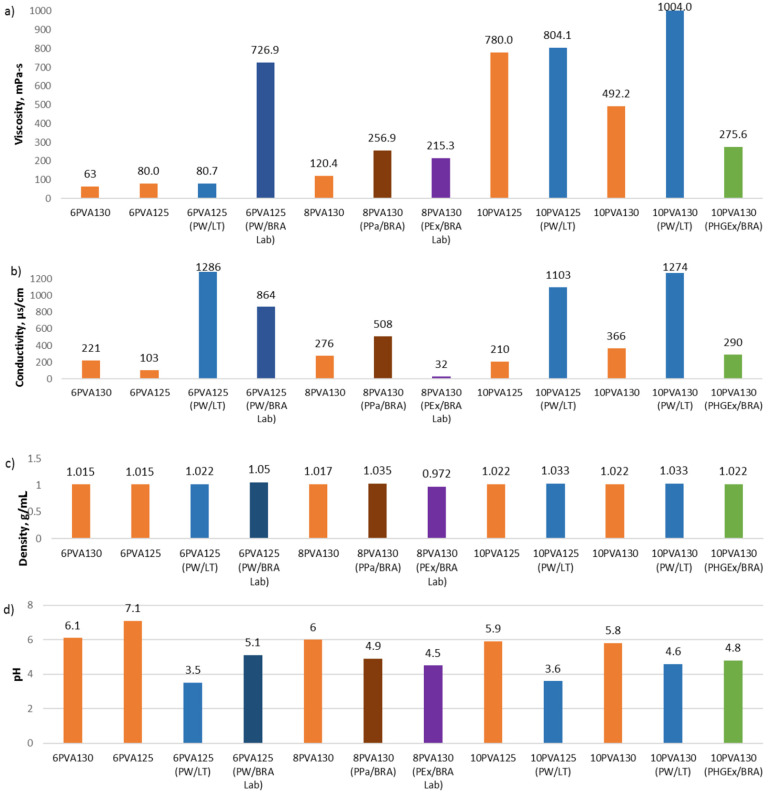
PVA and PVA with propolis water, propolis particles, hydroglyceric, or ethyl alcohol propolis extracts solutions’ properties: (**a**) viscosity, (**b**) conductivity, (**c**) density, and (**d**) pH.

We compared 6 wt% and 10 wt% PVA solutions to solutions to which propolis water has been added. As could be expected from the analysis of the properties, the addition of propolis water increases the viscosity, conductivity, and density for all samples (Figure 1). In particular, the viscosity of the 6PVA130 is close to a value of 60 mPa s, which is practically the lower limit of viscosity for spinning fibers with a cylindrical electrode. However, 6PVA125 viscosity is 80 mPa s, and PW/LT additive does not increase it substantially, but PW/BRA Lab increases viscosity dramatically up to 727 mPa s. The additive of PW/LT increases viscosity of 10PVA125 from 780 to 804 mPa s and 10PVA130 from 492 to 1004 mPa s. All solutions with the addition of propolis water have the appropriate viscosity for electrospinning in the range from 81 to 1004 mPa s (Figure 1a).

The PVA solutions 125 KDa and 130 kDa have the same density, i.e., 1.015 (6 w%) and 1.022 g/mL (10 wt%), respectively, and replacing distilled water with propolis water PW/LT increases the density to 1.022 g/mL (6PVA125 and 6PVA130) and 1.033 g/mL (10PVA130 and 10PVA125), and replacing distilled water with PW/BRA Lab increases the density to 1.05 g/mL (6PVA125). (Figure 1c).

Propolis water significantly increases the electrical conductivity of the spinning solutions (Figure 1b). The 6PVA126 conductivity increases from 103 to 1286 µS/cm when PW/LT additive is used and to 864 µS/cm when PW/BRA Lab is used. The conductivity of 10PVA125 increases from 210 to 1103 µS/cm and 10PVA130 from 366 to 1274 µS/cm when distilled water is replaced with PW/LT. All prepared solutions, with and without propolis water, have sufficient electrical conductivity to form fibers. 

The acidic environment of the propolis additives also significantly reduces the total pH of the spinning solutions but does not reach pH 2, which could significantly affect the fiber size (Figure 1d). 

Hydroglyceric propolis additive (PHGEx/BRA) decreases the viscosity of the 10PVA130 solution by 44% (Figure 1a) and the pH from 5.9 to 4.8 (Figure 1d), but the density does not change (Figure 1c) because the densities of the additive and the PVA solution are the same. The electrical conductivity also decreases from 366 to 290 μs/cm, but this still is sufficient for electrospinning (Figure 1b).

The addition of 3.85 wt% propolis powder (PPa/BRA) increases the viscosity, electrical conductivity, and density of 8PVA130 solution but decreases the pH from 6 to 4.9 (Figure 1).

Ethyl alcohol propolis extract (PEx/BRA Lab) additive increases the viscosity of the 8PVA130 solution and significantly reduces conductivity, but the density decreases under the influence of alcohol (Figure 1a–c). The propolis powder and alcohol extract decrease the pH correspondingly to 4.9 and 4.5 (Figure 1d).

### 2.3. Nanoweb Morphology

**Figure 2:**
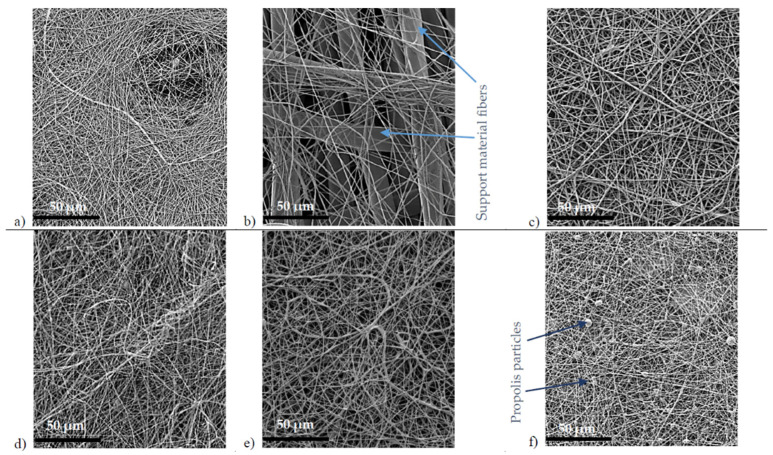
Micrographs of electrospun webs: (**a**) 10PVA130; (**b**) 10PVA130(PW/LT); (**c**) 10PVA130(PHGEx/BRA), (**d**) 8PVA130, (**e**) 10PVA130(PHGEx/BRA+ PEx/LV), (**f**) 8PVA130(PPa/BRA).

All propolis-containing solutions were spun stably, and no web defects such as film areas were visible. As mentioned in Section 2.2, the addition of propolis water increased the viscosity for all samples and the resulting fiber diameters. The only exception is hydroglyceric propolis extract/PVA solution, which turned into flat fibers instead of cylindrical ones, and the fiber width was not as homogeneous as that of the other samples due to the high glycerol content. The web of 10PVA130 (Figure 2a) shows that the fibers’ average diameter was of 286 ± 10 nm, the addition of propolis water (Figure 2b) increased the diameters to 664 ± 11 nm, hydroglyceric propolis extracts (Figure 2c) increased them to 606 ± 11 nm, and combination of hydroglyceric propolis extracts and propolis alcohol extract increased them to 491 ± 8 nm. The sample 10PVA130(PHGEx/BRA+ PEx/LV) fibers tended to form bundles; 2–6 fibers were stuck together, glued in the parallel direction (Figure 2e). The web of 8PVA130 (Figure 2d) showed fibers with an average diameter of 255 ± 4 nm, and the micrograph of sample 8PVA130 (PPa/BRA) shows inclusions of spherical propolis powder particles not exceeding 7000 nm in size (Figure 2f) and fibers with an average diameter of 218 ± 4 nm (Table 4).

### 2.4. Total Flavonoids and Propolis Content of Nanowebs

The flavonoids content of fibers containing propolis water PW/LT (4.74–4.96 mg/g) is close to the calculated flavonoid content (4.42–4.96 mg/g). It allows a relatively accurate prediction of the flavonoids content in the fibers and shows that propolis is present not only in the spinning solution but also in the same amount in the fibers. The content of propolis in the fibers is 73.5 % (Table 5) and may be higher if a 6 wt% PVA concentration solution is used. 

With the addition of propolis powder, it is possible to obtain fibers with a propolis content of 33%. In future studies, combining propolis water and propolis powder has the potential to achieve even higher propolis content in the fibers. The flavonoids content of fibers containing propolis particles PPa/BRA (8.3–8.88 mg/g) is lower than calculated (12.86–13.2 mg/g), which means that fewer propolis particles from the solution enter the web, such that it is possible that the solution is too saturated with propolis particles (Table 5).

The flavonoids content of fibers containing propolis glycerol extract PHGEx/BRA (4.91–5.34 mg/g) is slightly higher than calculated (4.49–5.17 mg/g), but it still allows a relatively accurate prediction of total flavonoid content in the fiber web. As glycerol acts as a plasticizer, and in view of SEM micrographs showing the formation of flat fibers (Figure 2c), the 7 wt% amount of hydroglyceric propolis extract in the 10PVA130 spinning solution results in only 12% propolis content in fibers (Table 5) and should not be exceeded. The hydroglyceric propolis extract datasheet indicates that it contains 20–25% propolis, but a concentration of 20% was used for the calculation, which may explain the slightly higher content of flavonoids, and the content of propolis in the fibers may reach to 15% if the 25% extract concentration is used.

The addition of propolis hydroglycerol extract and alcoholic extract in a 10 wt% PVA spinning solution allows to incorporate 26% of propolis in the electrospun fibers (Table 5), there are potential to add a higher amount of alcohol extract and reduce the PVA concentration to 8 wt% to achieve a higher propolis concentration in the electrospun fibers (depending on solution viscosity). The flavonoids content of fibers containing propolis extracts PHGEx/BRA and PEx/LV (32.72–33.54 mg/g) is a bit higher than calculated (30.33–31.01 mg/g), and the fiber web containing glycerol extract also had a slightly higher flavonoid content than expected, so the presence of glycerol in this sample is also likely to be due to a slightly higher actual flavonoid content than calculated.

### 2.5. Solution Stability and Shelf Life 

The sample 6PVA125(PW/LT) is homogeneous and can be spun even after 6 months (Figure S1a). The solution 6PVA125(PW/BRA Lab) precipitated 5 min after mixing with a magnetic stirrer (Figure S1c), and 3 h ultrasound mixing temporarily improved uniformity and particle size reduction, but the solution was also stratified after 5 min (Figure S1d–f). Therefore, premixing is required. The samples 10PVA125(PW/LT) and 10PVA130(PW/LT) have excellent stability of solutions: their spinnability time is 6 months and premixing is not required, nor was precipitate or mold observed.

After the solution 8PVA130(PPa/BRA) had settled for a week, a small precipitate was visible (Figure S2b). Additional ultrasonic mixing was also used to both reduce the particle size and improve the dispersion (Figure S2c–f). After the solution was mixed with a magnetic stirrer, large agglomerates (6–59 µm) were visible in the micrographs (Figure S2a), but after ultrasonic treatment for 3 h, their size decreased, and the largest particles were 2–12 µm (Figure S2d–f). No improvement was seen with processing longer than 3 h. After two weeks of storage at room temperature, mold appeared on the surface of the solution 8PVA130(PPa/BRA).

The shelf life of the solution 10PVA130(PHGEx/BRA) (yellow, transparent, clear liquid) is approximately 6 months at room temperature (Figure S3), after which visual changes are visible. The solution was still spinnable but premixing was needed.

In the solution 8PVA130(PEx/BRA Lab), the propolis particles were of different sizes and required sonication to decompose the propolis agglomerates. A small precipitate of propolis was visible after 1 h, and a clear precipitate of propolis was visible after 60 days (Figure S4), but spinnability time was only 1 week.

The 10PVA130(PHGEx/BRA+PEx/LV) sample with propolis alcohol extract and hydroglyceric propolis extract spun more stable than without glycerol, and its shelf life was significantly increased (1 week to 3 months (Figure S3)).

The stability of the solutions and shelf life are summarized in Table 6. Photographs and optical microscope images of the obtained solutions are shown in Figures S1–S4.

## 3. Materials and Methods

### 3.1. Materials

**Figure 3:**
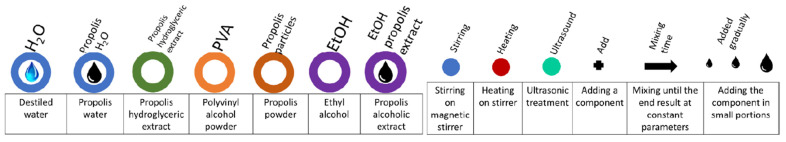
The components of electrospinning solutions and designations of solution-mixing process stages.

The polyvinyl alcohol polymer matrix used in this research was obtained from Sigma-Aldrich Chemical Company (Darmstadt, Germany). The series of samples using different PVA molecular weights Mowiol 20–98 (Mw = ~125 kDa, degree of hydrolysis 98.0–98.8) and polyvinyl alcohol 18–88 (Mw = ~130 kDa, degree of hydrolysis 86.7–88.7) were performed; the melting point was 200 °C and the density was 1.269 g/cm^3^. The total flavonoids content was determined according to [23], using zirconium oxychloride (98%, Mw = 322.25, Sigma-Aldrich) and methanol (99.9%, Mw = 32.04%, Sigma-Aldrich). The ethyl alcohol (96.4–96.6%, Mw = 46.07, SIA Kalsnavas Elevators, Jaunkalsnava, Latvia) used to prepare the laboratory-made propolis extracts. The extract from Riga Pharmaceutic Factory (Riga, Latvia) contains 70% ethyl alcohol extract. Various types of propolis and its extracts were used (Table 7). The stages of the solution-mixing process are designated as marked (Figure 3). Laboratory distilled water (conductivity 1 µS/cm) was used to prepare PVA solutions and propolis extracts.

### 3.2. Characterizations of Fiber Webs and Solutions

To assess the suitability of the prepared solutions for electrospinning, the density (using an aerometer), conductivity (conductometer Greisinger GLF-100, Regenstauf, Germany), viscosity (Viscosiometer Viscolead adv, Fungilab S.A.,100 rpm, used tip L6), and pH (pH/ORT Tester AD14, ADWA Instruments, Bucarest, Hungary) of each solution and propolis extract were determined at 20 ± 0.15 °C. The homogeneity of the spinning solutions was analyzed by Stereo Zoom Microscope (Motic SMZ-171, Xiamen, China). Electrospun Fibers quality and diameters were analyzed from micrographs obtained by scanning electron microscope (SEM Mira Tescan, Bern, Czech Republic), before covered with a gold coating (5–20 nm). The image analysis program ImageJ was used for diameters measurements (at least one hundred measurements at five different locations for each sample). Total flavonoid contents in propolis and fiber webs were determined with a UV-VIS spectrophotometer (Perkin Elmer Lamda 650, Waltham, MA, USA). 

### 3.3. Propolis Extracts Preparation

**Figure 4:**
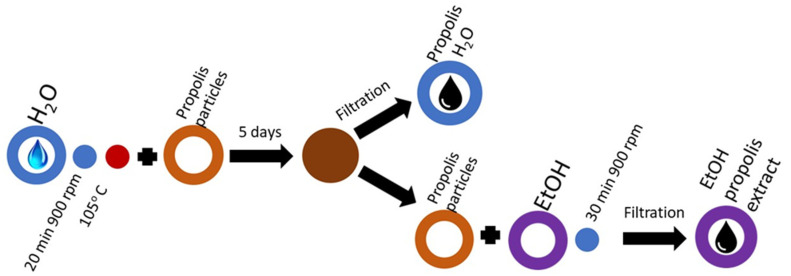
Mixing sequence required for laboratory-made propolis water (PW/BRA Lab) and ethyl alcohol propolis extract (PEx/BRA Lab) preparation.

To prepare the laboratory-made propolis water, 20 g of powder is added to 200 mL of water and boiled for 20 min to remove the Arabic Gum or beeswax. The proportions of water and propolis are in accordance with the requirements for the determination of beeswax in propolis [24]. Further, the solution is filtered through Nr. 398 cellulose filter and allowed to stand for 5 days. The liquid with the resin is poured off, and propolis water PW/BRA Lab solution is obtained (PW/LV Lab samples are prepared with the same method using raw propolis with a wax content of 25%, which was separated after boiling). The propolis placed at the bottom of the vessel is poured with ethyl alcohol and stirred for 30 min to obtain PEx/BRA Lab extract; see mixing sequence Figure 4. Propolis extracts PW/BRA Lab and PEx/BRA Lab are made from PPA/BRA powder. Propolis extracts PW/LV Lab and PEx/LV Lab are made from PPA/LV raw propolis according to Figure 4.

### 3.4. Determination of Flavonoids 

The 1 mL of propolis or electrospun web sample (in 0.01 % methanol) was placed in two parallel tubes and 7 mL of methanol was added to one tube. In a second tube, 1 mL of 2% ZrOCl_2_ 8H_2_O and 6 mL of methanol were added. The solutions were mixed, and after 1 h, the absorbance was measured at 436 nm. Total flavonoids were calculated as the routine equivalent of the calibration graph and expressed as mg routine/g sample.

### 3.5. Solutions Preparation for Electrospinning

**Figure 5:**
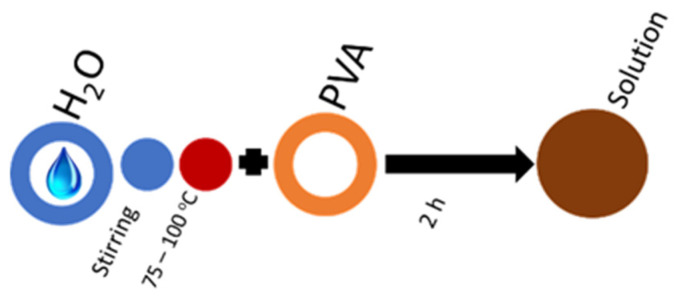
The mixing sequence required for PVA electrospinning solutions.

For each PVA molecular-weight solution, concentrations 6, 8, and 10 wt% were used; stirring time, mixing temperature (Table 8), and mixing sequence are shown in Figure 5. The solutions require 2 h of stirring at 1000 rpm on a magnetic stirrer (BioSan MSH-300, Riga, Latvia) to prepare 60 mL of spinning solution.

### 3.6. Propolis Integration

**Figure 6:**
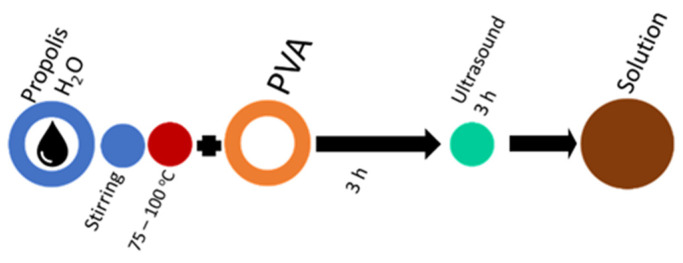
Solution mixing sequence for propolis water integration into electrospinning solution.

**Figure 7:**
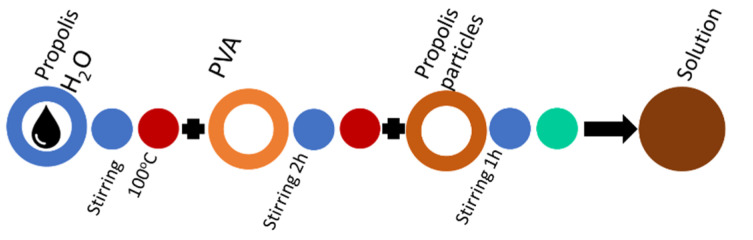
Solution mixing sequence for propolis water and propolis powder integration.

**Figure 8:**
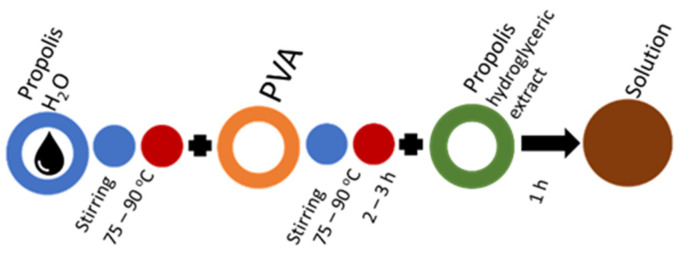
Solution mixing sequence for propolis water and propolis hydroglyceric extract integration.

**Figure 9:**
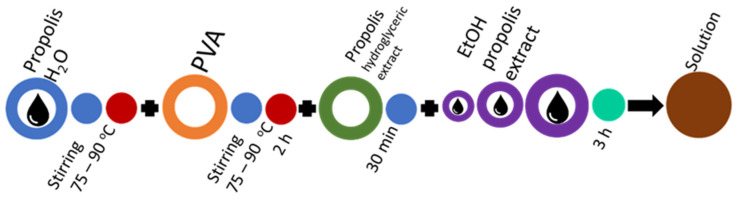
Solution mixing sequence for propolis water and propolis ethyl alcohol extract integration.

In the basic mixing scheme of the PVA solution (Figure 5), distilled water is replaced by propolis water. This mixing sequence is suitable for industrially produced propolis water, but laboratory-made propolis water also requires sonication to break up the particles that have passed through the filter and prevent the solution from stratification and to prevent from air inclusions (Figure 6). Propolis particles can be integrated with propolis water (Figure 7). Propolis particles dispersed in PVA solution were treated with high-intensity ultrasonic treatment (ultrasonic processor UP 200 H, 200 W, frequency 26 kHz, amplitude 90%, sonotrode S26, Ø14 mm) for 3 h. In order to control the process temperature, the solution was put in an ice-water bath due to propolis’s low melting point (it usually melts between 80 and 105 °C). If propolis hydroglyceric extract is needed, it can be added only when the PVA is completely dissolved. The total preparation time using 130 kDa PVA does not exceed 3 h (Figure 8). If propolis water contains a precipitate, pre-sonication is required before it is mixed with PVA. The addition of the propolis alcohol extract in one step with water leads to larger agglomerates than the addition once the PVA has dissolved, as the solubility of the propolis decreases as the concentration of ethyl alcohol decreases (propolis alcohol integration strategies, see also Figure S4). It is important to stir the solution rapidly and add the propolis extract to the cooled solution gradually. The sequences shown in Figure 8 and Figure 9 can be combined by first adding propolis hydroglyceric extract to the PVA solution and then the ethyl alcohol extract.

The 4 solutions with 90–94 wt% propolis water (PW) content and one solution with propolis powder with 3.85 wt% additive (PPa/BRA), propolis hydroglyceric extract 7 wt% (PHGEx/BRA), and propolis ethyl alcohol extract 7 wt% (PEx/BRA Lab) were obtained to evaluate the effect of the propolis additive on solution viscosity, conductivity, and pH. A solution with a combination of propolis alcohol 7 wt% and hydroglyceric extract 7 wt% was also prepared (Table 9).

### 3.7. Solution Shelf Life and Stability Evaluation

After integration of the propolis in solution, it was divided into two parts. One part was spun, but the other part was allowed to stand at room temperature for 6 months. Every week, changes were inspected visually. As soon as a change was observed, the solution was electrospun to determine the spinnability. If there was a precipitate, premixing was performed.

### 3.8. Fabrications of Nanowebs

The cylinder-type electrode (Ø 20 mm, length 150 mm, 2/3 immersed into solution bath) was used to obtain nanowebs by using electrospinning equipment (Nanospider Lab 200 from Elamarco, Liberec, Czech Republic). The electrospinning voltage was 55–62 kV, the distance between the electrodes was 18 cm, and the electrode rotation speed 4 rpm was adjusted according to the molecular weight of each polymer and the concentration of PVA in the solution. Electrospinning took place at 21.5 °C and relative air humidity ranged from 18 to 20%. Polypropylene nonwoven was used as a support material for fiber collection.

## 4. Conclusions

Various propolis preparations were integrated into PVA electrospinning solutions, resulting in high-quality fiber webs that would benefit productivity and industrial applications. The obtained fiber webs could be used in biomedicine and bioprotective systems, and the material consists of edible ingredients, extending its applications.

Various propolis integration strategies using different propolis additives allow for the incorporation of more than 73% propolis into the fibers. The combination of propolis water, hydroglyceric propolis extract, and ethyl alcohol propolis extract can increase propolis content even more. The highest propolis content in the fibers can be integrated using propolis water (73.5 wt%) and the highest flavonoid content was achieved using a combination of ethyl alcohol and glycerol extracts (33.13 ± 0.41 mg/m). Using 130 kDa PVA allows propolis extracts to be incorporated using lower mixing temperatures and times than 125 kDa PVA. Both molecular weights are suitable for the integration of propolis at a concentration of not less than 6 wt% because the viscosity is sufficient. The only exception is a 6 wt% 130 kDa PVA solution, with insufficient viscosity to add ethyl alcohol extracts, so the 8 wt% concentration is recommended.

By determining the flavonoids content of propolis extracts (which allows predicting bioactivity), it is also possible to predict the flavonoid content in the fibers. Solutions for electrospinning with propolis water and hydroglyceric propolis extract have a shelf life of up to 6 months at room temperature, but premixing is required. The addition of hydroglyceric propolis extract improves the shelf life and spinnability of solutions that are less stable. For solutions with propolis particles integrated as a powder or with alcoholic extract, it is better to spin them immediately. The most suitable PVA molecular weights for propolis integration are 125 kDa and 130 kDa when 6 wt% is used as the lowest concentration and the samples are spun with roller-type equipment using cylindrical electrode.

## Figures and Tables

**Table 1:** Propolis water samples properties.

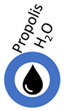	**Sample**	**PW/LT**	**PW/LV Lab**	**PW/LV**	**PW/BRA Lab**
	pH	3.6	3.4	4.3	4.6
	Density, g/mL	1.010	0.995	1.000	1.032
	Conductivity, µS/cm	1526	359	352	1400
	Total flavonoids, mg/g	0.087 ± 0.005	0.11 ± 0.01	0.051 ± 0.003	1.25 ± 0.05
	Flavonoids to propolis, mg/g	0.0003	0.0022	0.0003	0.0139
	Propolis content, %	30.8	5	15	10

**Table 2:** Properties of propolis powder and hydroglyceric propolis extracts.

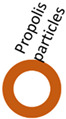	**Sample**	**PPa/BRA**	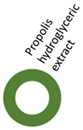	**Sample**	**PHGEx/BRA**
	Powder particle size, µm	<200 *		pH	4.5
	Density, g/mL	0.8 ± 0.05		Density, g/mL	1.220 *
				Conductivity, µS/cm	3
	Total flavonoids, mg/g	5.43 ± 0.07		Total flavonoids, mg/g	1.15 ± 0.08
	Propolis content, %	~100		Propolis content, %	20–25 *

* Data from data sheets.

**Table 3:** Properties of ethyl alcohol propolis extracts.

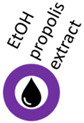	**Sample**	**PEx/LV Lab**	**PEx/LV**	**PEx/BRA Lab**
	pH	4.9	5.3	5.2
	Density, g/mL	0.885	0.905	0.886
	Conductivity, µS/cm	9	41	57
	Total flavonoids, mg/g	8.87 ± 0.05	5.75 ± 0.05	7.53 ± 0.09
	Propolis content, %	30	30	30

**Table 4:** Fibers diameters.

Nanoweb Sample	Diameters, nm	Median	Mode
8PVA130	255 ± 4	252	252
8PVA130 (PPa/BRA)	218 ± 4	218	213
10PVA130	286 ± 10	271	214
10PVA130 (PW/LT)	664 ± 11	652	657
10PVA130 (PHGEx/BRA)	606 ± 11	609	490
10PVA130 (PHGEx/BRA+PEx/LV)	491 ± 8	489	471

**Table 5:** Flavonoids and propolis content in fibers.

	Electrospinning Solution, 60 g		Fibers			
Nanoweb Sample	Calculated Propolis Content g	PVA, g	Web Weight, g	Calculated Propolis Content, %	Total Flavonoids Content mg/g	Calculated Flavonoids Content mg/g
10PVA130(PW/LT)	16.63	6	22.63	73.5	4.85 ± 0.11	4.698 ± 0.27
8PVA130(PPa/BRA)	2.4	4.8	7.20	33.33	8.59 ± 0.29	13.03 ± 0.17
10PVA130(PHGEx/BRA)	0.84	6	6.84	12.28	5.13 ± 0.21	4.83 ± 0.34
10PVA130(PHGEx/BRA+PEx/LV)	2.12	6	8.12	26.11	33.13 ± 0.41	30.67 ± 0.34

**Table 6:** Stability and shelf life of electrospinning solutions with propolis additive.

Nanoweb Sample	Stability of the Solution	Precipitate	Spinnability Time Limit	Molding	Premixing Required
6PVA125(PW/LT)	Excellent	-	6 months	-	Yes
6PVA125(PW/BRA Lab)	Bad	5 min	6 months	-	Yes
10PVA125(PW/LT)	Excellent	-	6 months	-	-
10PVA130(PW/LT)	Excellent	-	6 months	-	-
8PVA130(PPa/ BRA)	Average	1 week	1 week	After 2 weeks	Yes
10PVA130(PHGEx/BRA)	Good	-	6 months	-	Yes
8PVA130(PEx/BRA Lab)	Bad	1 h	1 week	-	Yes
10PVA130(PHGEx/BRA+PEx/LV)	Average	1 h	3 months	-	Yes

**Table 7:** Data of propolis and its extracts.

	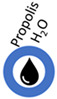				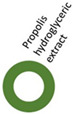	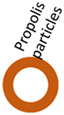		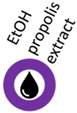		
**Solvent**	**Water**				**Glyceric**	**-**		**Etilalcohol**		
**Propolis Type**	PW/LT	PW/ LV	PW/ LV Lab	PW/ BRA Lab	PHGEx/ BRA	PPA/ BRA	PPA/ LV	PEx/LV	PEx/ LV Lab	PEx/ BRA Lab
**Manufacturer**	Medicata	Deiva	Laboratory-made		B Natural	B Natural	Bee keepers	Riga Pharm. Factory	Laboratory-made	
**Propolis Content**	30.8%	15%	5% *	10% *	20–25%	100% **	100% **	30%	3% *	30% *
**Propolis Origin**	Lithuania	Latvia	Latvia	Brazil	Brazil	Brazil	Latvia	Latvia	Latvia	Brazil

* The content of propolis in laboratory-made extracts is determined by weighing five samples after evaporation of the solvent and calculating the average weight; ** may contain Arabic Gum or waxes.

**Table 8:** The time and temperature used for PVA solutions preparation.

Sample	Molar Weight, kDa	PVA Content in Solution, wt%	Mixing Temp., °C	Stirring Time, h
6PVA125	125	6	90–100	2
8PVA125		8		
10PVA125		10		
6PVA130	130	6	75–90	
8PVA130		8		
10PVA130		10		

**Table 9:** Electrospinning solutions with propolis additives.

Sample	Temp., °C	Time, Hours Stirring/ Ultrasound	Propolis Additive Content in Solution, wt%	PVA Content in Solution, wt%
6PVA125(PW/LT)	100	3	94	6
6PVA125(PW/BRA Lab)		5/3	94	6
10PVA125(PW/LT)		3	90	10
10PVA130(PW/LT)	75–90	2	90	10
8PVA130(PPa/ BRA)	100	3/3	3.85	8
10PVA130(PHGEx/BRA)	75–90	3	7	10
8PVA130(PEx/BRA Lab)		2.5/3	7	8
10PVA130(PHGEx/BRA+PEx/LV)		2.5/3	7 + 7	10

## Data Availability

The data presented in this study are available in this article and supplementary materials.

## Supplementary Materials

The following supporting information can be downloaded at: https://www.mdpi.com/article/10.3390/molecules27072311/s1, Table S1: Electrospinning solutions designations and contents; Table S2: Data of propolis and its extracts; Table S3: Data of propolis particle size after various mixing cycles of spinning solution 8PVA130(PPa/BRA) *; Figures S1–S4: Photographs and optical microscope images of the obtained solutions.

## Sample Availability

Samples of the compounds are available from the corresponding author.
