# Supplementary material for: Propolis Integration Methods into Solutions for Highly Loaded Propolis Fibers by Needleless Electrospinning
Source: Molecules. 2022 Apr 2;27(7):2311. doi: 10.3390/molecules27072311 (PMC9000478; doi:10.3390/molecules27072311)
Supplement: Supplementary file 1 [file molecules-27-02311-s001.zip › molecules-1640709-supplementary.pdf]

Supplementary Materials

Propolis Integration Methods into Solutions for Highly Loaded Propolis Fibers by Needleless Electrospinning

Zane Zelča [1,*], Silvija Kukle [1], Sarmīte Janceva [2], Laimdota Vilcēna [1]

[1] Institute of Design technology, Faculty of Materials Science and Applied Chemistry, Riga Technical University, Latvia;
[2] Latvian State Institute of Wood Chemistry, Latvia;
* Corresponding author – Zane.Zelca@rtu.lv, Riga Technical University, Kipsala street 6, Riga, Latvia

The homogeneity of the spinning solutions was analyzed by stereo zoom microscope (Motic SMZ-171); 40x and 100x magnifications were used.

Table S1. Electrospinning solutions designations and contents.

Sample	Propolis Additive Content in Solution, wt%	PVA Content in Solution, wt%
6PVA125(PW/LT)	94	6
6PVA125(PW/BRA Lab)	94	6
10PVA125(PW/LT)	90	10
10PVA130(PW/LT)	90	10
10PVA130(PHGEx/BRA)	7	10
8PVA130(PPa/ BRA)	3.85	8
8PVA130(PEx/BRA Lab)	7	8
10PVA130(PHGEx/BRA+PEx/LV)	7 and 7	10

Samples are marked with polyvinyl alcohol concentration and molecular weight (kDa), and propolis extract in parentheses.

Table S2. Data of propolis and its extracts.

	Propolis Water		Propolis Particles	Hydroglyceric Extract	Alcoholic Extract	
**Propolis type**	**PW/LT**	**PW/ BRA Lab**	**PPA/ LV**	**PHGEx/ BRA**	**PEx/ BRA Lab**	**PEx/LV**
**Manufacturer**	Medicata	Laboratory-made	Bee keepers	B Natural	Laboratory-made	Riga Pharm. Factory
**Propolis content**	30.8%	10%*	100% **	20–25%	30% *	30%
**Propolis origin**	Lithuania	Brazil	Latvia	Brazil	Brazil	Latvia

* The content of propolis in laboratory-made extracts was determined by weighing five samples after evaporation of the solvent and calculating the average weight; ** May contain Arabic Gum or waxes.

The solutions require 2 h of stirring at 1000 rpm with a magnetic stirrer (BioSan MSH-300) to prepare 60 ml of spinning solution. PVA solutions were treated with high-intensity ultrasonic treatment (ultrasonic processor UP 200 H, 200 W, frequency 26 kHz, amplitude 90 %, sonotrode S26, Ø14 mm).

Table S3. Data of propolis particle size after various mixing cycles of spinning solution 8PVA130(PPa/ BRA) *

Mixing	Magnetic stirrer	Ultrasonic treatment for 1 h	Ultrasonic treatment for 3 h
Particle size, μm	6–59	3–46	2–12

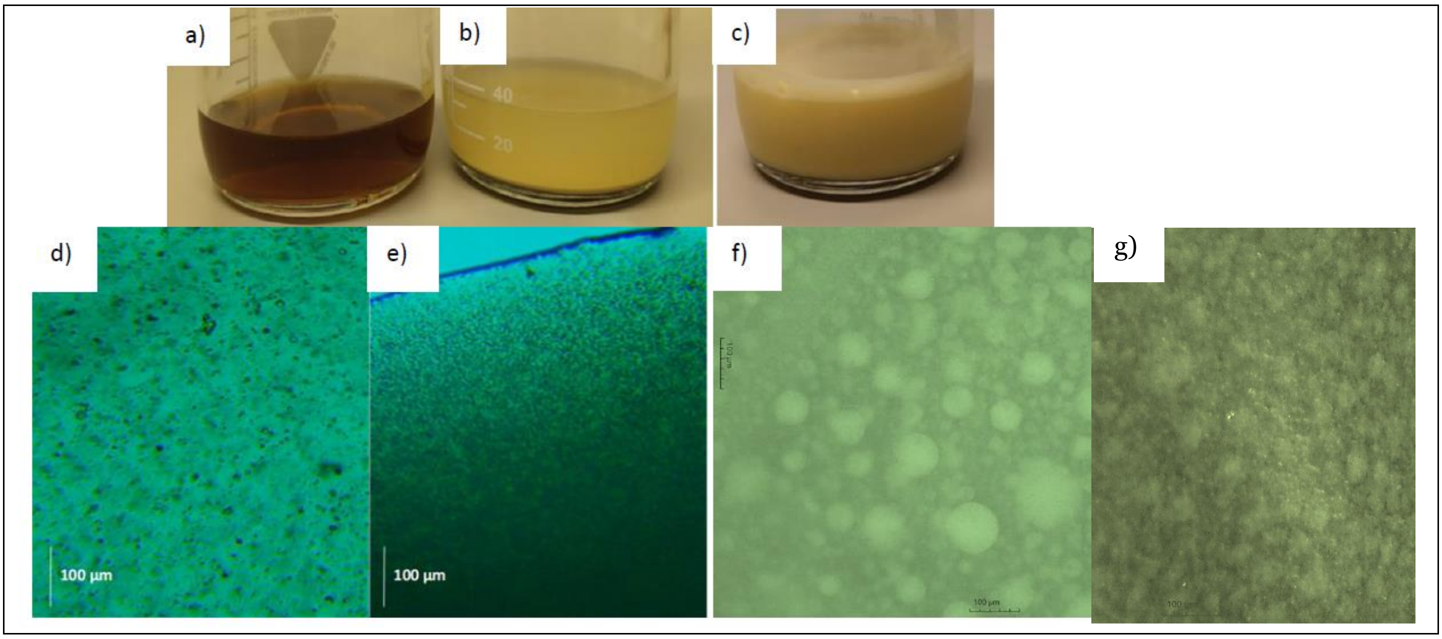

Figure S1. Photographs and optical microscope images of the obtained solutions: **a**) 6PVA125(PW/LT); **b**) 6PVA125(PW/BRA Lab) 5 min after mixing; **c**) 6PVA125(PW/BRA Lab) just mixed; **d**) 6PVA125(PW/BRA Lab) mixed 1h with ultrasound; **e**) 6PVA125(PW/BRA Lab) mixed 3h with ultrasound; **f**) 6PVA125(PW/BRA Lab) after 1 week; **g**) 6PVA125(PW/BRA Lab) mixed for 3h with ultrasound and settled for 5 min.

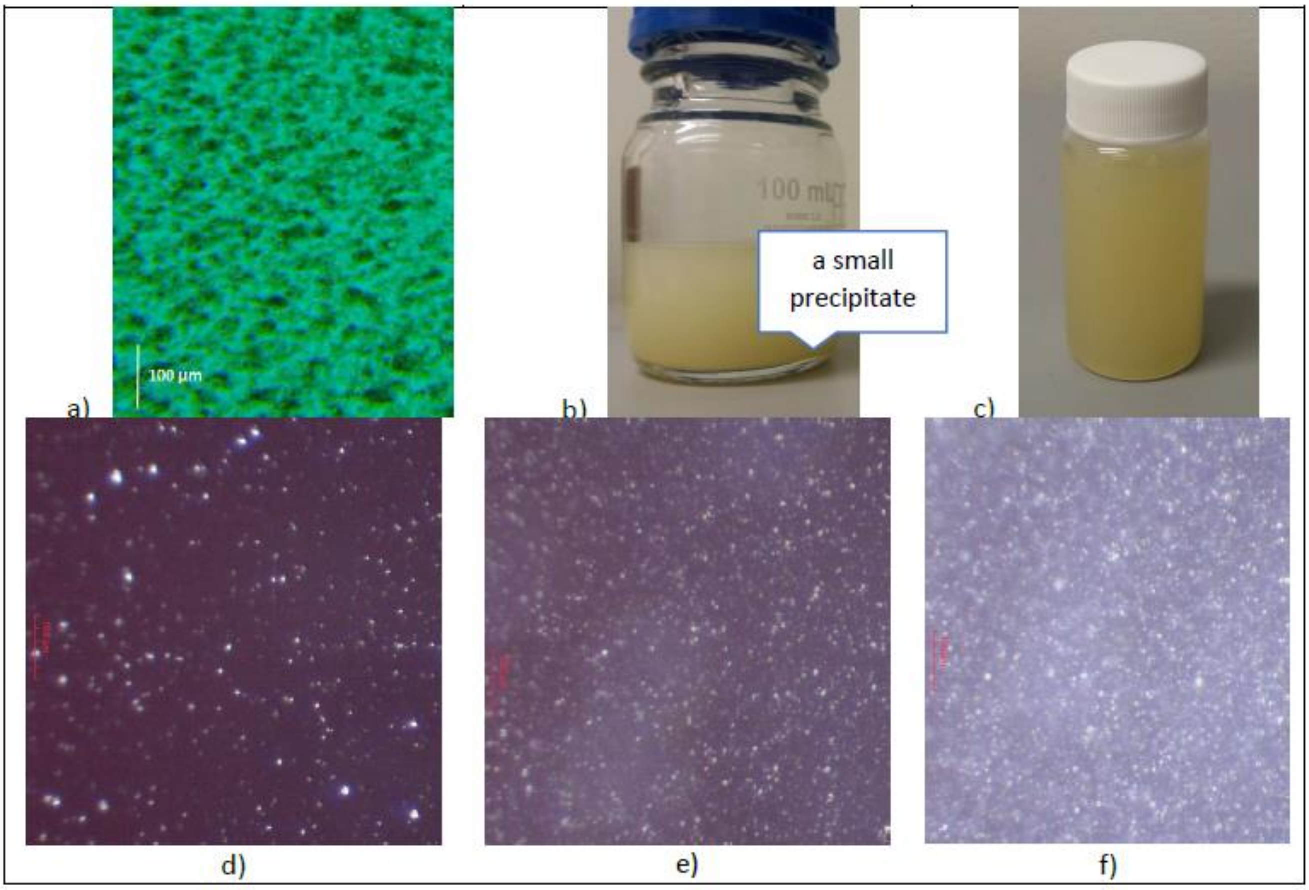

Figure S2. Photographs and optical microscope images of the obtained solutions: 6PVA125(PPa/BRA) just mixed with magnetic stirrer (**a**) and settled 1 month (**b**,**d**) and mixed with ultrasound 1h (**d**) and 3h (**c**, **f**).

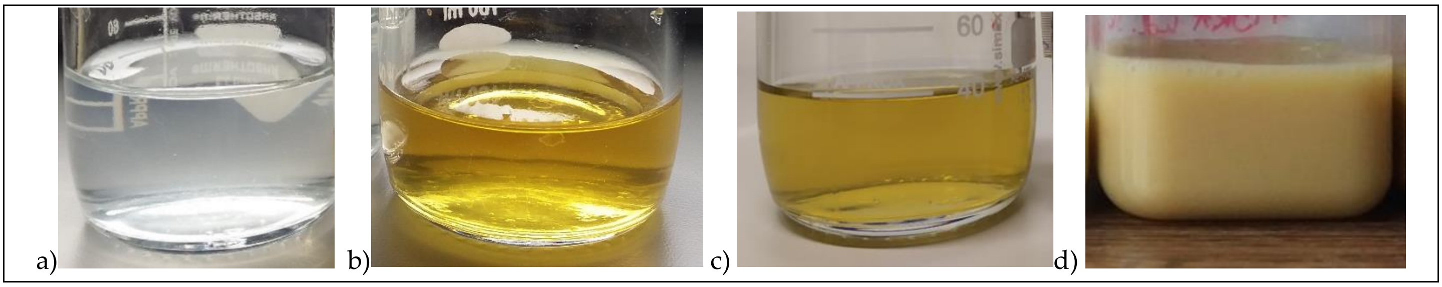

Figure S3. Photographs of the obtained solutions: 10PVA130 (**a**), 10PVA130(PHGEx/BRA) just mixed with magnetic stirrer (**b**), 10PVA130(PHGEx/BRA) settled for 6 months (**c**), 10PVA130(PHGEx/BRA+PEx/LV) settled for 3 months (**d**).

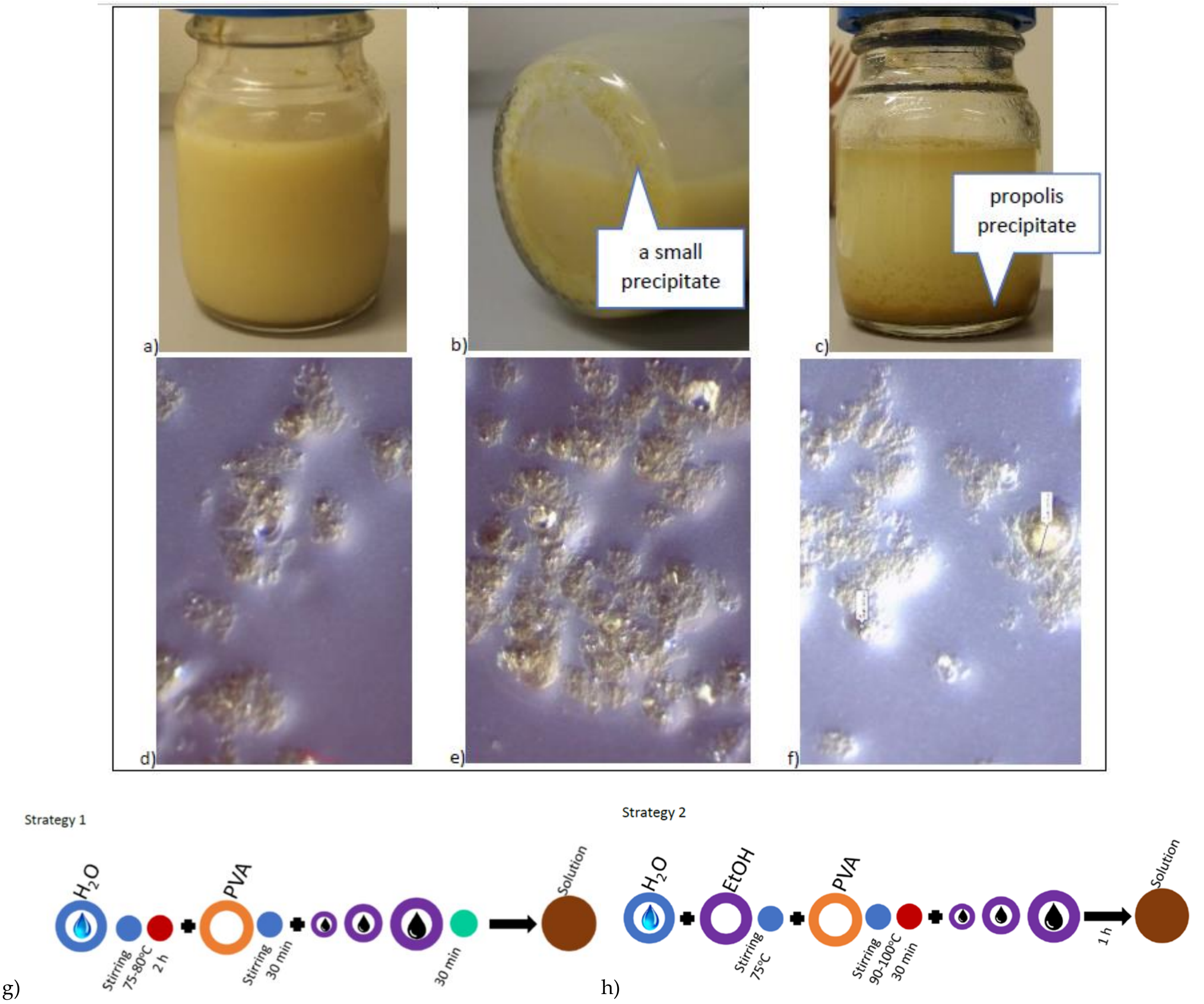

Figure S4. Photographs and optical microscope images of the obtained solutions: 8PVA130(PEx/BRA Lab) just mixed with magnetic stirrer (**a**) and settled 5 days (**b**) and settled for 60 days (**c**), propolis solution at optical microscope 100x magnification (**d**-**f**); Propolis ethyl alcohol extract integration strategies into 8 wt% PVA solution. Strategy 1: addition of the extract to the finished PVA solution (**g**); Strategy 2: Dissolution of PVA in aqueous / alcoholic solution and subsequent addition of propolis extract.
